# Assessment of Extracellular Particles Directly in Diluted Plasma and Blood by Interferometric Light Microscopy. A Study of 613 Human and 163 Canine Samples

**DOI:** 10.3390/cells13242054

**Published:** 2024-12-12

**Authors:** Boštjan Korenjak, Armando Tratenšek, Matevž Arko, Anna Romolo, Matej Hočevar, Matic Kisovec, Maxence Berry, Apolonija Bedina Zavec, David Drobne, Tomaž Vovk, Aleš Iglič, Alenka Nemec Svete, Vladimira Erjavec, Veronika Kralj-Iglič

**Affiliations:** 1 University of Ljubljana, Faculty of Health Sciences, Laboratory of Clinical Biophysics, SI-1000 Ljubljana, Slovenia; bostjan.korenjak@zf.uni-lj.si (B.K.); matevz.arko@zf.uni-lj.si (M.A.); anna.romolo@zf.uni-lj.si (A.R.); maxence.berry@etu.univ-poitiers.fr (M.B.); 2 University of Ljubljana, Faculty of Pharmacy, SI-1000 Ljubljana, Slovenia; armando.tratensek@ffa.uni-lj.si (A.T.); tomaz.vovk@ffa.uni-lj.si (T.V.); 3 Institute of Metals and Technology, SI-1000 Ljubljana, Slovenia; matej.hocevar@imt.si; 4 National Institute of Chemistry, SI-1000 Ljubljana, Slovenia; matic.kisovec@ki.si (M.K.); polona.bedina@ki.si (A.B.Z.); 5 College for Basic and Applied Sciences, University of Poitiers, 86000 Poitiers, France; 6 Department of Gastroenterology, University Medical Centre Ljubljana, SI-1000 Ljubljana, Slovenia; david.drobne@kclj.si; 7 University of Ljubljana, Faculty of Medicine, SI-1000 Ljubljana, Slovenia; 8 University of Ljubljana, Faculty of Electrical Engineering, Laboratory of Physics, SI-1000 Ljubljana, Slovenia; ales.iglic@fe.uni-lj.si; 9 University of Ljubljana, Veterinary Faculty, Small Animal Clinic, SI-1000 Ljubljana, Slovenia; alenka.nemecsvete@vf.uni-lj.si (A.N.S.); vladimira.erjavec@vf.uni-lj.si (V.E.)

**Keywords:** extracellular particles, extracellular vesicles, exosomes, membrane vesiculation, platelets, blood products, platelet-rich plasma, liquid biopsy, membrane enclosed, drug delivery

## Abstract

Extracellular nanoparticles (EPs) are a subject of increasing interest for their biological role as mediators in cell–cell communication; however, their harvesting and assessment from bodily fluids are challenging, as processing can significantly affect samples. With the aim of minimizing processing artifacts, we assessed the number density (*n*) and hydrodynamic diameter (*D*_h_) of EPs directly in diluted plasma and blood using the following recently developed technique: interferometric light microscopy (ILM). We analyzed 613 blood and plasma samples from human patients with inflammatory bowel disease (IBD), collected in trisodium citrate and ethylenediaminetetraacetic acid (EDTA) anticoagulants, and 163 blood and plasma samples from canine patients with brachycephalic obstructive airway syndrome (BOAS). We found a highly statistically significant correlation between *n* in the plasma and *n* in the blood only in the human (i.e., but not canine) blood samples, between the samples with trisodium citrate and EDTA, and between the respective *D*_h_ for both species (all *p* < 10^−3^). In the human plasma, the average <*D*_h_> was 139 ± 31 nm; in the human blood, <*D*_h_> was 158 ± 11 nm; in the canine plasma, <*D*_h_> was 155 ± 32 nm; and in the canine blood, <*D*_h_> was 171 ± 33 nm. The differences within species were statistically significant (*p* < 10^−2^), with sufficient statistical power (P > 0.8). For <*n*>, we found no statistically significant differences between the human plasma and blood samples or between the samples with trisodium citrate and EDTA. Our results prove that measuring *n* and *D*_h_ of EPs in minimally processed fresh blood and in diluted fresh plasma by means of ILM is feasible for large populations of samples.

## 1. Introduction

Blood and plasma contain cells, soluble proteins, such as albumin, immunoglobulin, fibrinogen, lipoprotein, and ribonucleoprotein aggregates, and nano-sized extracellular particles (EPs). In 1976, a report on harvesting platelet-derived EPs presented electron microscope images of EPs and of activated platelets undergoing budding [1]. It was indicated that EPs could have biological effects, thereby implying their potential roles in diagnostics and therapy [1]. Since then, numerous studies on blood EPs have been performed, and knowledge of EPs’ physicochemical properties, morphology, and specific biological effects has accumulated ([2,3,4] and included references). Here, we focus on ex vivo blood EPs and plasma EPs.

EPs mediate intercellular communication by exchanging the material and information stored within cellular fragments [3,4]. They travel to a recipient cell where the EPs’ cargoes (including proteins and various types of nucleic acids and their fragments) can be internalized. By activating remote cellular processes, EPs become the effectors of systemic metabolic regulation [5]. In samples, EPs are considered potential indicators of the body’s clinical status, in particular, its response to disease. They have been isolated from diverse bodily fluids, including cerebrospinal fluid, breast milk, semen, urine, saliva, amniotic fluid, ascites, bile, and blood [3], as well as from plants [6].

The basic required information about EPs includes the identity (indicated by the shape and ultrastructure), number density (*n*), and size in vivo. However, with currently available methods, blood EPs cannot be assessed in vivo, and sampling should be performed prior to their observation and characterization. The most widely used methods are (differential) ultracentrifugation (UC), size-exclusion chromatography (SEC), filtration, precipitation, and capture by affinity structures [4].

EPs are relatively small particles (smaller than 1 μm, down to tens of nanometers). Because of resolution limits, they cannot be visualized live using an optical microscope. Scanning electron microscopy (SEM) [7,8,9,10] and transmission electron microscopy (TEM) [11,12,13,14] provide information on the identity, shape, and size of EPs. Cryogenic TEM (cryoTEM) also shows the ultrastructure of EPs [15,16]. In samples, EPs are numerous, and batch methods provide information on the population of EPs in a sample. Flow cytometry (FCM) is used to assess the number density of EPs (*n*) and their distribution [17,18,19]. Dynamic light scattering (DLS) provides the mean hydrodynamic diameter of EPs (*D*_h_); the dispersity index and the intensity of scattered light that accounts for the abundance of EPs [20,21] and, in combination with static light scattering, yields the radius of gyration (*R*_g_), which enables the determination of the structural factor *R*_g_/*R*_h,0_ (where *R*_h_ = *D*_h_/2) [22,23]. Nanotracking analysis (NTA) [24,25] and interferometric light microscopy (ILM) [26,27,28,29] are used to determine both *n* and *D*_h_. Tunable resistive pulse sensing (TRPS) [30,31,32,33] is based on changes in the electric current in which the EPs are participating and yields their *n* and size. Nanostructural information on highly monodispersed and concentrated EP samples can be acquired by small-angle X-ray scattering (SAXS) [34].

Batch methods are unable to distinguish different types of EPs in samples that have similar sizes and physical properties but differ in their origins and compositions [35]. Furthermore, experimental evidence indicates that the methods used to harvest and assess EPs significantly influence the content of samples [36]. To obtain plasma from blood, erythrocytes are sedimented, usually by means of centrifugation. Although the centripetal acceleration of the centrifuge rotor required to produce plasma is moderate, blood cells are affected [37]. Blood manipulation may promote cell vesiculation, vesicle fragmentation, or fusion, as well as interactions with other particles in a sample and with the material in contact with the sample. This can affect the configuration of a sample. It is likely that in blood preparations, the populations of particles acquired and measured using different methods are, to a large extent, diverse. This is particularly detrimental for their potential use in clinics, as information about the characteristics of the samples that may reflect the clinical status of the blood donor can be altered because of processing.

A possible aspect would be to avoid processing samples as much as possible. In light of this aim, FCM was applied to assess *n* of EPs directly in diluted blood from 22 healthy subjects [38], and in 56 patients with pancreatic cancer and 48 healthy controls [39]. Marchisio et al. (2021) sorted fluorescent-dyed EVs according to their markings [38], and Brocco et al. (2022) related *n* to the clinical status of the patients [39]. EPs were assessed in plasma without prior isolation—by DLS [20,40], NTA [35,41], TRPS [41,42], and FCM [43]. Božič et al. (2019) assessed the average *D*_h_ of EPs in diluted and filtered plasma samples from three healthy donors [22]. Kogej et al. (2021) applied the method proposed by Božič et al. (2019) to diluted and filtered plasma samples from seven patients with ovarian cancer, seven controls, and two patients with other diseases [23]. In a pilot study, Berry et al. (2024) measured *D*_h_ of EPs in 250 diluted plasma samples from multiple species (canine, equine, and human) by ILM [44].

The methods that show promise for clinical applications should prove feasible for larger populations of samples. Following agreement between the results obtained by DLS and ILM on a small number of various samples [28] and in a preliminary multispecies study [44], in this work we report on the assessment of *n* and *D*_h_ by ILM in the following two larger populations involved in ongoing clinical studies: human patients with IBD and canine patients with BOAS.

## 2. Materials and Methods

Information regarding the sample preparation is documented in the MIBlood-EV reports.

### 2.1. Human Patients

The observational study included 74 patients (35 females and 39 males) diagnosed with inflammatory bowel disease (39 with Crohn’s disease and 35 with ulcerative colitis). The median age of the cohort was 46 years (interquartile range [IQR] 31–59 years), the median height was 172 cm (IQR 165–179 cm), and the median weight was 72 kg (IQR 62–84 kg). The study participants were outpatients at the University Medical Center Ljubljana, starting a new biological treatment at the time of inclusion in the study. Patients were followed for 6 months, during which multiple samples were collected for analysis, including 166 samples of plasma with trisodium citrate and 154 samples of each of the following: plasma with EDTA, blood with trisodium citrate, and blood with EDTA (altogether 613 samples). The study was approved by the National Medical Ethics Committee of the Republic of Slovenia (0120-271/2022/4; KME 27 July 2022).

### 2.2. Canine Patients

The study was conducted at the Small Animal Clinic of the Veterinary Faculty, University of Ljubljana, Slovenia, from March 2023 to August 2024. Seventy-six privately owned brachycephalic dogs were included in the study. The inclusion criteria were as follows: no coexisting systemic diseases, no medical treatment or vaccination within the previous month, a diagnosis of brachycephalic obstructive airway syndrome (BOAS), and no previous upper airway surgery. The diagnosis of BOAS was based on clinical signs and clinical examination and confirmed with an endoscopic examination of the upper airways. All dogs included in the study presented with severe multilayer upper airway obstruction and were classified as BOAS+ [45]. Patients were then followed for one year, during which, for some dogs, another set of samples was collected. Eighty-eight samples of each of the following were included: plasma with EDTA and blood with EDTA (altogether 176 samples). Formal written informed consent was obtained from the owners before the dogs participated in the study. All procedures in the study complied with the relevant Slovenian government regulations (Animal Protection Act, Official Gazette of the Republic of Slovenia, No. 43/2007). The study was approved by the Animals in Experiments Welfare Commission of the Veterinary Faculty, University of Ljubljana, approval number: 18-3/2022-1.

### 2.3. Blood Sampling in Human Patients

Blood was collected in the morning hours during regular ambulatory visits. A G21 needle (Microlance, Becton Dickinson, Plymouth, UK) and a 3.6 mL evacuated tube with trisodium citrate (Vacutube^®^, 9NC, LT Burnik, Ltd., Komenda, Slovenia) or 6 mL evacuated tube with potassium EDTA (BD Vacutainers^®^, 367864, Becton Dickinson, Plymouth, UK) were used. Samples were then transported to the laboratory (by bicycle (ca. 3 km)) where plasma preparation and dilution of the plasma and blood took place.

### 2.4. Blood Sampling in Canine Patients

Collection occurred in the morning after fasting for a minimum of 15 h overnight. A G21 needle (Microlance, Becton Dickinson, Franklin Lakes, NJ, USA) and 2 mL evacuated tubes with EDTA (Vacuette, Greiner Bio-One, Kremsmunster, Austria) were used. Blood was transported to a laboratory located in the room next to the one in which the blood sampling took place to prepare the plasma, diluted plasma, and diluted blood.

### 2.5. Preparation of Human Plasma and Blood

A total of 100 μL of the trisodium citrate blood was removed and placed into an Eppendorf tube. The rest of the sample was centrifuged to obtain plasma. To sediment the erythrocytes, blood was centrifuged at room temperature for 10 min at 1480× *g* (centrifuge Centric 322A, Domel, Železniki, Slovenia). The supernatant from above the buffy coat was collected in an Eppendorf tube to constitute a 200 μL plasma aliquot. The same procedure was used for the preparation of the potassium EDTA samples. Blood and plasma samples were transported to the laboratory (by bicycle (ca. 1 km)) where the measurements by means of ILM took place.

### 2.6. Preparation of Diluted Canine Plasma and Blood

A total of 5 μL of EDTA whole blood was pipetted into 995 μL of saline (200× dilution). The rest of the EDTA whole blood was centrifuged to obtain plasma; to sediment the erythrocytes, the blood was centrifuged for 15 min at 1500× *g* and 21 °C (centrifuge Heraeus Megafuge 8R, Thermo Fisher Scientific, Osterode Am Harz, Germany). The supernatant from above the buffy coat was collected into Eppendorf tubes to constitute 250 μL plasma aliquots. A total of 4 μL of EDTA plasma was pipetted into 196 μL of saline (50× dilution). Plasma, diluted plasma, and diluted blood samples were transported to the laboratory (by car (ca. 1 km)) where the measurements by means of ILM took place.

### 2.7. Interferometric Light Microscopy (ILM)

Samples were processed within 4 h from the sampling. The average *n* and *D*_h_ of the EPs were determined by means of ILM using Videodrop (Myriade, Paris, France). If convenient, samples were additionally diluted with physiologic saline (B. Braun Melsungen AG, Melsungen, Germany) that was kept in sealed containers at room temperature—to reach the optimal number density of EPs for detection (between 5 × 10^8^ and 5 × 10^9^ particles per mL in the sample). Measurements were performed at room temperature. The signals of the saline were under the detection limit. A total of 7 μL of sample was placed between cover glasses and illuminated by a 2 W blue LED light. A threshold value of 4.2 was used. The light shed on the sample and the light scattered on the particles was imaged using a bright-field microscope objective. Images of the interference between the incident and scattered light were captured using a complementary metal–oxide–semiconductor high-resolution, high-speed camera and analyzed. The signal of the incident light was subtracted from the image. Patterns that included contrasting black and white spots were recognized as particles. The number density of the particles was estimated as the ratio between the number of detected particles and the volume scanned (e.g., 15 pL). The movement of every particle was recorded on video. The *D*_h_ of a particle was determined by tracking the position of the imaged particle in the recorded video. Each video consisted of 100 frames recorded at 140 frames per second (lasting less than one second). Several successive videos were made, and the time that elapsed between two videos was about 15 s. It is estimated that about 90% of the nanoparticles undergoing Brownian motion in a typical volume imaged by ILM move out of that volume in about 0.45 s. Consequently, the nanoparticle population is almost completely renewed in each video. If a nanoparticle was detected in a different frame within the video, its track was reconstructed. The diffusion coefficient (D) of the motion of the particle was taken proportional to the mean square displacement (*d*) of the particle between two consecutive recorded frames taken in the time interval ∆*t*, <*d*^2^(∆*t*)> = <4D ∆*t*> (where the parentheses <> mark the averaging). The *D*_h_ value was estimated by assuming that the particles were spherical and using the Stokes–Einstein relation *D*_h_ = *kT*/3πηD. A limit of 10 movies or 300 detected particles was set for each sample. Processing of the images and of the movies was performed using the associated software QVIR 2.6.0 (Myriade, Paris, France).

### 2.8. Scanning Electron Microscopy of Plasma (SEM)

A total of 20 microliters of sample was placed on a 50 nm carbon filter in the incubation chamber, and 40 microliters of 1% OsO_4_ solution was poured on top. After incubation for 1 h, the solution was pushed through the filter. The filter was removed from the incubation chamber and placed in a well filled with distilled water. The filter was incubated in water for 10 min, repeated 3 times. Then, the filter was dehydrated in a graded series of ethanol (30%, 50%, 70%, 80%, 90%, and 100%, 10 min each) and hexamethyldisilazane mixed with absolute ethanol (30%, 50%, and 100%, 10 min each) and, finally, air-dried. The dried sample was sputtered with a mixture of gold and palladium and examined with a scanning electron microscope (JSM-6500F, JEOL Ltd., Tokyo, Japan).

### 2.9. Cryogenic Transmission Electron Microscopy (cryoTEM)

Aliquots of 250 μL of plasma were placed in Eppendorf tubes and centrifuged for 10 min at 17,570× *g* and at room temperature in a Centric 200R centrifuge with a Lilliput rotor (Domel, Železniki, Slovenia). The upper 200 μL of supernatant was replaced with saline, resuspended, and centrifuged again for 10 min at 17,570× *g* and room temperature in the Centric 200R centrifuge with Lilliput rotor (Domel, Železniki, Slovenia). A total of 200 μL of supernatant was removed, and the pellet gathered from the aliquots was suspended in 80 μL of saline for imaging with CryoTEM Vitrobot Mark IV (Thermo Fisher Scientific, Waltham, MA, USA). The C-flat (2 µm hole size, 2 µm hole spacing, 200 mesh holey carbon grids (EMS, Hatfield, PA, USA)) were glow discharged for 60 s at 20 mA and positive polarity in an air atmosphere (GloQube^®^ Plus, Quorum, Laughton, UK). The conditions were set at 4 °C, 95% relative humidity, blot time: 7 s, and blot force: 2. Three μL of the sample with nanoparticles in suspension was applied to the grid, blotted, and vitrified in liquid ethane. Samples were visualized under cryogenic conditions using a 200 kV Glacios microscope with a Falcon 3EC detector (Thermo Fisher Scientific, Waltham, MA, USA).

### 2.10. Statistical Analysis

All measurements were performed in triplicate and are presented as the average value and standard deviations. The number of samples included in the final analysis was lower than the number of samples acquired, as for some samples, the number density of EPs was under the detection limit or technical issues occurred in their transport and handling. The number of samples included in the assessment of each parameter is provided with the results. Correlations between variables were assessed using the Pearson correlation coefficient (*r*) and the respective probability (p). A value of p = 0.05 was taken as the threshold for statistical significance. Differences between samples were determined by the *t*-test and respective probability (p), with a value of p = 0.05 taken as the threshold for statistical significance. For statistically significant differences, statistical power was calculated using Web-based Sample Size/Power Calculations; Inference for Means: Comparing Two Independent Samples https://www.stat.ubc.ca/~rollin/stats/ssize/n2.html (accessed on 28 October 2024). The value of statistical power P > 0.8 was taken as sufficient.

## 3. Results

**Figure 1:**
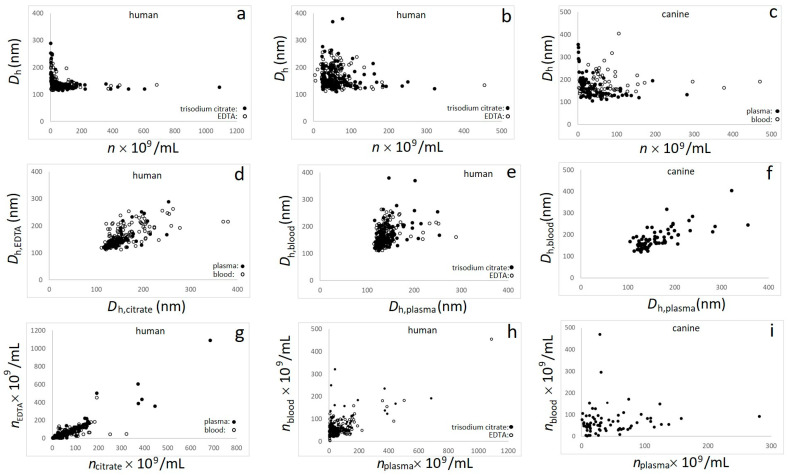
(**a**) Hydrodynamic diameter of EPs *D*_h_ as a function of the number density of EPs *n* in human plasma; (**b**) *D*_h_ as a function of *n* in human blood; (**c**) *D*_h_ as a function of *n* in canine plasma and blood; (**d**) agreement between *D*_h_ of EPs in the samples collected in two anticoagulants (trisodium citrate and EDTA); (**e**) agreement between *D*_h_ of EPs in human plasma and in human blood; (**f**) agreement between *D*_h_ of EPs in canine plasma and in canine blood; (**g**) agreement between *n* in samples collected in two anticoagulants (trisodium citrate and EDTA); (**h**) agreement between *n* in human plasma and in human blood; (**i**) agreement between *n* in canine plasma and in canine blood.

**Figure 2:**
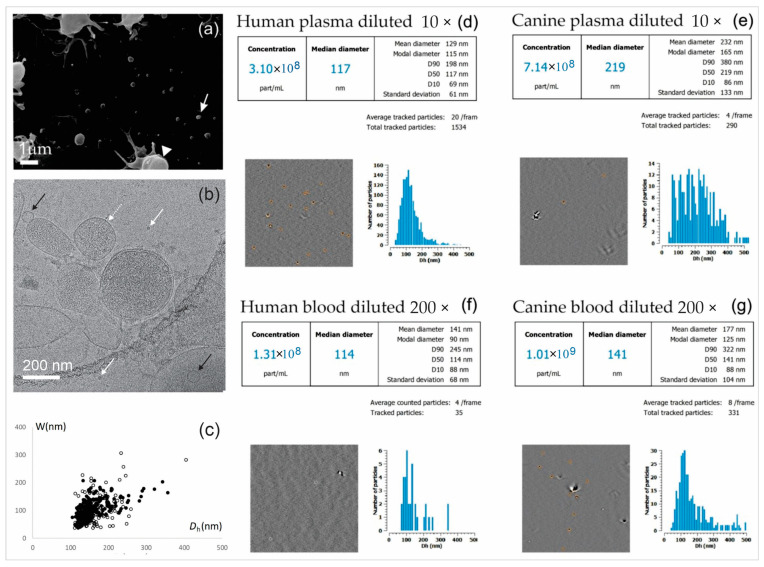
(**a**) Scanning electron micrograph of the canine plasma showing EPs (white arrow) and platelets (white triangle); (**b**) cryogenic transmission electron micrograph of EPs isolated from human plasma showing membrane-enclosed vesicles of various sizes (dashed white arrow points to a large vesicle, and black arrows point to smaller vesicles) and molecular complexes (white arrows); (**c**) correlation between *D*_h_ and the width of the distribution *W* for all analyzed samples (full circles: plasma; empty circles: blood); (**d**–**g**) examples of ILM results of human plasma and blood and of canine plasma and blood showing the distribution over *D*_h_ and displaying the detected particles (orange circles).

The raw data used to compose Figure 1 and Figure 2, as well as Table 1, Table 2, Table 3 and Table 4, are provided in the Supplementary Materials. Figure 1 shows the interdependencies between *n* (corrected for dilution) and *D*_h_ in human plasma, human blood, canine plasma, and canine blood. In human samples, two anticoagulants were considered (trisodium citrate and EDTA). It can be seen that at higher values of *n*, the *D*_h_(*n*) dependence saturated to an expected constant value; however, it increased considerably with a decrease in *n* (Figure 1a–c). This was observed for all groups of samples, but it was more notable for plasma than blood (Figure 1a and black circles in Figure 1c). The measurements in blood exhibited higher scattering over the whole range of *n* (Figure 1b and empty circles in Figure 1c) which masked the effect.

The correlations between the assessed *D*_h_ in the human plasma with trisodium citrate and *D*_h_ in the human plasma with EDTA, as well as between *n* in the human plasma with trisodium citrate and *n* in the human plasma with EDTA, were strong and the dependencies exhibited the expected 1:1 slope (Figure 1d,g), suggesting that at the population level the choice of the anticoagulant did not have a detectable effect on the results. The *D*_h_ in human plasma and *D*_h_ in human and canine blood were strongly correlated; however, the slopes indicated larger EPs in blood than in plasma (Figure 1e,f). We found a statistically highly significant correlation between *n* in plasma and in blood in human (Figure 1h) but not in the dog (Figure 1i).

The Pearson coefficients of the various correlations are presented in Table 1. It can be seen that all correlations, but correlation between *n* in the canine plasma and *n* in the canine blood, were highly statistically significant (*p* < 10^−4^). The strongest correlations were found for the human plasma between the two anticoagulants in *n* (*r* = 0.94; Table 1 and Figure 1g) and *D*_h_ (*r* = 0.77; Table 1 and Figure 1d), as well as between *n* in human plasma and blood with EDTA anticoagulant (r = 0.81; Table 1 and Figure 1h). It can be seen that the Pearson coefficients could reach values higher than 0.9, which sets a high standard for the achievable level of quality for assessments via ILM.

We considered (see Section 4: Discussion) that the steep increase in *D*_h_ with a decrease in *n* at low *n* is partly an artifact. In a further statistical analysis, we therefore placed confidence in the results for the region of *n* where *D*_h_ had saturated and assumed that the *D*_h_ values of a great majority of EPs in vivo are smaller than 250 nm. Because of the above arguments regarding the quality of the measurements (Figure 1a–c), we discarded samples with *D*_h_ larger than 250 nm in estimation of the population averages <*n*> and <*D*_h_>.

The <*D*_h_> values in the samples with trisodium citrate did not differ from the <*D*_h_> values in the samples with EDTA both in the plasma and blood (Table 2). In both the human and canine samples, <*D*_h_> in blood was higher than that in plasma (Table 2). The <*D*_h_> value in the canine samples was higher than that in the human samples, for both plasma and blood (Table 2). All statistically significant differences, but the difference between the canine plasma and canine blood, were of sufficient statistical power (P ≥ 0.8).

The average number densities <*n*> of EPs in human plasma with trisodium citrate and with EDTA, in human blood with trisodium citrate and with EDTA, and in human blood and plasma with trisodium citrate showed no statistically significant differences (Table 3). The difference between <*n*> in the canine plasma and blood was statistically significant with sufficient statistical power (Table 3).

The recommended optimal range for the measured *n* of the ILM equipment was between 0.5 × 10^9^/mL and 5.0 × 10^9^/mL. Most plasma samples were within this interval (<*n*> (uncorrected for dilution) in human plasma was 1.30 × 10^9^/mL in trisodium citrate, and 1.31 × 10^9^/mL in EDTA and in canine samples it was 1.29 × 10^9^/mL (Table 4)). In blood, the measured values of <*n*> were considerably smaller (0.22 × 10^9^/mL in both trisodium citrate and EDTA in human samples, as well as 0.30 × 10^9^/mL in canine samples) (Table 4), which is out of the recommended optimal range, but still notably higher than the signal of the saline which was always lower than 0.1 × 10^9^/mL. The blood samples were more diluted to enable saturation of light which was expected to be absorbed in the residual cells. The range of dissipation of the number densities (*n*) in the blood samples was larger in blood than in plasma (Figure 1a–c), which is reflected in the respective standard deviations that are comparable to the average values (Table 3 and Table 4).

In ILM, each particle is tracked by recording a video and analyzed individually. It follows from Table 4 that more than 270,000 EPs have been assessed in human samples and more than 70,000 in canine samples.

Figure 2 shows a SEM image of canine plasma and a cryoTEM image of EPs isolated from human plasma. For the cryoTEM, the cells had to be removed from the sample by means of filtering. Figure 2a shows EPs (white arrow) and residual activated platelets (white triangle) with tubular protrusions. EPs are globular and heterogeneous in size. Figure 2b shows membrane-enclosed particles of different sizes, in agreement with Figure 2a. The width of the distribution over *D*_h_ for each sample is represented by the standard deviation of the distribution (*W*). Figure 2c shows the correlation between *D*_h_ and *W* for all analyzed samples. The Pearson correlation coefficient is 0.63 in plasma and 0.48 in blood; both correlations are highly statistically significant (*p* < 10^−5^). Examples of the distributions are presented in Figure 2d–g. In the human plasma and blood examples (Figure 2d and Figure 2f, respectively), *D*_h_ is small and the distribution is narrow (i.e., the reported standard deviation is small). In the canine examples, the distributions are wider, and the average values of *D*_h_ are higher (Figure 2e,g). In the interference micrographs, identified EPs are marked with orange circles. The presence of singular, larger particles can be noted.

## 4. Discussion

### 4.1. Need of Sufficient Population Size for Translational Medicine

EPs derived from blood are of the utmost interest for diagnostics, monitoring, and therapeutics, as they may travel via circulation and reach areas that are not directly accessible in an intact body. Since Wolf (1967) indicated their important biological role [1], EPs have been the subject of extensive study [2,3,4]. However, in spite of their potential involvement in basic cellular processes and the vast amount of accumulated data, to the best of our knowledge, methods based on blood-derived EPs have not yet made a breakthrough into everyday clinical practice. This proves that translation of basic EV research into clinical practice is a challenging task [5,39].

In analyses of small populations, it is questionable whether the cases are representative and whether the results are based on differences between the populations or on the noise caused by the limitations of the methods. Clinical studies involving EPs are often limited by a relatively small number of cases reflecting lengthy and/or sophisticated harvesting procedures, too small a yield of EPs—even to apply multiple assessment techniques—and insufficient repeatability of the procedures. A meta-analysis that included 716 comparisons between populations of EP samples [46] reported that 308 (43%) comparisons had statistically significant differences (p < 0.05). Sufficient statistical power (P > 0.8) was found in 242 (34%) and clinical significance, estimated as a modified Reliable Change Index larger than 1.96 (less than 5% false-positive and/or false-negative results) [46]), was found only in 88 (12%). None of the studies amid the 12% included more than 50 cases within both populations [46]. Pursuing decisive answers regarding clinically relevant questions must be based on methods that enable the analysis of sufficiently large populations of samples. Our results prove that analysis of diluted blood and diluted plasma with ILM enables the determination of basic information (number density and size of EPs and their hydrodynamic diameter) in large cohorts of samples.

### 4.2. Less Is More in Sample Processing

To obtain plasma, erythrocytes are removed, and this is usually performed by centrifugation of blood. The movement of blood cells during centrifugation is rather complex and may be a source of EV shedding (particularly from platelets) [47]. Platelets are prone to fragment because of shear stress which develops during sampling and centrifugation [37,48,49]. In principle, to minimize platelet activation and fragmentation, sampling of blood should be as gentle as possible regarding the shear stress; shaking of samples (e.g., during transport) should be avoided, and it would be best to process the samples immediately at the same location where the blood was sampled.

It was recently reported [47] that during erythrocyte sedimentation, plasma is pushed in the opposite direction, thereby carrying platelets, EPs, and molecules toward the top of the tube. In this phase, the supernatant becomes enriched in platelets and small particles. However, when the lower bound of the plasma meets with the upper bound of the erythrocytes, further centrifugation sediments also the platelets and, to a certain extent, the EPs, so plasma becomes poorer with these constituents [47]. The process depends on the physical properties of the blood and on the geometry of the tubes and centrifuge rotor. The shear forces acting on the platelets depend on the viscosity of the medium, which is in turn sensitive to the temperature. Taking blood out of the body exerts thermic stress on the cells [36]. Centrifugation of blood is usually performed in refrigerated centrifuges that have a feedback loop to level the temperature in the rotor chamber by turning on the cooling when the temperature, which increased because of the friction of the bearings, exceeds the required value. The timing of this occurrence is not repeatable and may strike the samples in different particle distribution configurations. Usually, plasma is prepared using the same centrifuge settings for all samples, although the properties of the samples are different and are affected by the same external parameters to a different degree. On the other hand, with respect to the randomness of the processing parameters described above, it would be best if the blood samples were centrifuged within the same lot. However, the number of samples that can be loaded into the centrifuge is rather small, and even with this number, some samples must wait. During isolation/enrichment and assessment procedures, particles in plasma are exposed to further mechanical stress or physicochemical modifications, which does not affect all samples evenly, as they have different compositions and physical properties. All of the above processes may differ considerably among individual samples.

It was reported that the presence of small amounts of particles up to 1 μm in size generally does not compromise the accuracy of the NTA measurements [50]. In DLS, the intensity of the scattered light is proportional to the square of the volume of the particle, which makes DLS very sensitive to the presence of large particles; small amounts of large aggregates or dust particles can disturb the size determination if the main population is significantly smaller in size [51]. Therefore, larger particles (e.g., cells) should be removed from the samples prior to the measurement. In isolation and characterization, the noise increases with each step. Omitting procedures in processing means the only possibility of avoiding certain sources of artefacts.

### 4.3. Characterization of Human IBD and Canine BOAS Populations

The principal aim of the IBD and BOAS clinical studies (albeit beyond the scope of this work) was to see whether EPs can be used as a parameter to follow-up on the effect of the treatment. The BOAS study was the first to start. The Small Animal Clinic at the Veterinary Faculty, University of Ljubljana, has skilled technicians and a well-equipped laboratory next door to the room where the blood sampling is performed. This was particularly advantageous, as previous work indicated the importance of avoiding any stress in transporting and saving the samples [37]. The staff had experience in blood sampling for EP assessment from a previous collaboration. Therefore, it seemed optimal to process the samples immediately after the sampling at the clinics. A small amount of blood was diluted and the rest was centrifuged to obtain plasma. As we did not have experience, we did not know what would be appropriate range of the dilution. The dilutions of plasma (50×) and blood (200×) were set based on previous results [47]. Moreover, the rest of the plasma was conserved to allow for different dilutions. These samples were transported by car to the laboratory for measurement with ILM (distance of about 1 km) and were assessed within 4 h of the sampling. On the other hand, the sampling of blood from patients with IBD took place at the University Medical Centre, Ljubljana. Samples were transported to the Faculty of Pharmacy (ca. 3 km away) by bicycle. A part of the blood was saved and another part centrifuged to produce plasma. Undiluted blood and plasma were transported by bicycle to the laboratory where appropriate dilutions of blood and plasma and measurement with ILM took place (distance of about 1 km). Although the design of the study and laboratory configuration was more favorable in the BOAS study, strong correlations were found between all parameters, except between *n* in canine plasma and blood (Table 1). It seems that the decisive difference between the two studies is in the dilution of the blood samples. In canine blood, the results fell within the range of a low *n*/large *D*_h_ region of *D*_h_(*n*) dependence that is subject to artefacts and large dissipation of the results (Figure 1c). Too high dilution and a uniform choice of dilution for all samples in the BOAS study turned out to be a disadvantage over the possibility to adjust the dilution to the individual sample (which took place in the IBD study). The sampling design, preparation of plasma, and dilution and transport of samples should be further improved also considering the individualization of plasma and blood preparation. However, the results derived from the parallel cohorts of samples were valuable, as such experiences point to possible limitations and accelerate the optimization of the methods. An evaluation of the correlation between EPs in plasma and blood is plausible and feasible, and it could be considered for use in assessing the quality of the processing.

### 4.4. Comparison with Studies in the Literature

Table 5 presents <*n*> and <*D*_h_> of EPs obtained in this study with <*n*> and size estimations from studies found in the literature. We performed a search according to keywords (e.g., plasma, blood, extracellular vesicles, extracellular particles, size, and concentration) in Google Scholar. We present a comparison of our results with 55 cohorts of EP samples (30 studies) using different preparations of plasma and different assessment techniques. We found two studies considering assessment of EPs directly in diluted blood by FCM [38,39]. Comparing <*n*> of EPs in diluted blood shows that the results obtained by FCM were four orders of magnitude smaller than our results (obtained by ILM), despite the sample preparation methods being similar (dilution of blood in PBS [39] or saline (this work)).

In the assessment of <*n*> directly in diluted plasma, the values spanned seven orders of magnitude—between 2 × 10^5^/mL (FCM [52]) and 5 × 10^12^/mL (NTA [35]). Isolation by UC yielded <*n*> between 0.27 × 10^9^/mL [58] and 603 × 10^9^/mL [57] (both NTA). Isolation by SEC yielded <*n*> between 10 × 10^9^/mL [64] and 4975.6 × 10^9^/mL [58] (both NTA), and precipitation/membrane-based affinity yielded results between 7.6 × 10^9^/mL [69] and 7383 × 10^9^/mL [57] (both NTA). Overall, the results presented in Table 5 span seven orders of magnitude, from 0.0002 × 10^9^/mL (diluted plasma, FCM) to 7383 × 10^9^/mL (precipitation, NTA). This observation exceeds the notion of Johnsen et al. (2019), who reported that the EV number density in plasma spans six orders of magnitude, depending on the harvesting and measurement methods [70]. The results presented in Table 5 confirm that <*n*> of EPs strongly depends on the sample preparation and assessment methods.

The method of harvesting the EPs is also important in the determination of the size, as different methods harvest different populations of particles. We estimated the size of the EPs directly in diluted blood by <*D*_h_> (146 nm), whereas Marchisio et al. (2021) estimated the size to be larger than 160 nm using FCM [38]. Directly in diluted plasma, the estimated <*D*_h_> were between 20 nm (DLS [23]) and 266 nm (DLS [20]). In isolates obtained using UC, the estimated <*D*_h_> were between 70 nm (NTA [35,56]) and 208 nm (NTA [57]). For SEC, the estimated <*D*_h_> were between 70 nm (TRPS [65]) and 202 nm (NTA [66]). Precipitation/membrane-based affinity yielded <*D*_h_> between 77 nm (DLS [57]) and 238 nm (NTA [55]). For TEM, the assessed sizes were between 87 and 112 nm [60]. Overall, the average estimated sizes were between 20 nm and 234 nm, which is a much smaller interval than the range of <*n*>, (0.00004–7383) × 10^9^/mL. In determining the size, the instruments limit the interval of the possible size results; however, in principle, sizes larger than 250 nm can be assessed by ILM, DLS, NTA, and TEM. Although the distributions of the individual measurements were subject to finite widths that were much larger than the standard deviations of the repeated measurements (e.g., Figure 2, panels (d)–(g)), in Table 5, all of the reported <*D*_h_> are smaller than 250 nm.

The batch techniques ILM, NTA, DLS, and TRPS generally do not distinguish different types of EPs in samples. It was suggested that lipoproteins are the main type of lipid particles in blood-derived samples [71,72,73,74]. The sizes of the lipoproteins assessed by nuclear magnetic resonance imaging of 27673 blood samples [75] were 21 nm for low-density lipoproteins, 9 nm for high-density lipoproteins, and 47–49 nm for very low-density lipoproteins. ILM does not detect particles smaller than about 80 nm (e.g., lipoproteins) and larger than about 500 nm (e.g., cells), which is well-suited for the detection of EPs. Because of their size range, lipoproteins do not interfere with the measurement of EPs using instruments with a threshold of approximately 80 nm [76]. However, lipoproteins may interact with EPs [72,73]. In addition, a protein corona can form on EPs [77,78], and complexes cannot be distinguished from undecorated EPs with existing batch methods [72,73,74]. Moreover, the proportion of lipoproteins in a sample may depend on the harvesting method [76,79,80]. György et al. (2010) pointed to the presence of immune complexes in samples and suggested using lysis of membrane-enclosed vesicles with detergent to quantify the respective populations [76].

### 4.5. Outlines and Perspectives

In spite of the expected high noise with harvesting and assessment procedures (as reflected in the large standard deviations), the measured and corrected values of <*n*> in the human plasma and blood were remarkably similar (Table 3 and Table 4). The samples in plasma and blood showed statistically significant correlations (Figure 1, Table 1), which indicates that a considerable portion of the EPs in the plasma also constituted the population of EPs in the blood—or were derived from EPs in the blood. This shows that the properties of the samples, not just the limitations of the instruments, contributed to the results. In the canine samples, we found no statistically significant correlation between *n* in the plasma and *n* in the blood; in our opinion, this was due to the too high blood dilution imposed for all canine blood samples, resulting in high levels of noise in the measurements of blood EPs. Accordingly, greater scattering in the *D*_h_(*n*) dependence in the canine blood (Figure 1c, empty circles) can be noted compared to the human blood (Figure 1b).

We found no statistically significant differences between respective <*n*> or <*D*_h_> in the populations of samples with the two anticoagulants (trisodium citrate and EDTA) (Table 2 and Table 3), neither in human plasma nor in human blood (Table 2), and the correlations between the respective parameters in the samples with trisodium citrate and EDTA were statistically significant (Table 1). This indicates that, at a population level, the choice between trisodium citrate and EDTA did not have an impact—as previously observed by Bettio et al. (2023) [55].

We found statistically significant differences with sufficient statistical power between <*D*_h_> in the plasma and blood in human and in the dog (Table 2). The EPs were, on average, larger in the blood than in the plasma (Table 2). All reported correlations of *D*_h_ were statistically significant.

Although our results obtained from diluted blood exhibit greater scattering than those in diluted plasma (Figure 1), we think that improvement in the preparation of samples (in particular, measurement directly in diluted blood) could enable a critical number of population studies to achieve sufficient statistical power and also clinical relevance—thereby paving the way for clinically relevant assessments of the number density and size of EPs. The dilution of blood seems to be the least detrimental of all procedures aimed at assessing the number density and size of EPs in blood. We outline the analysis of EPs in a multicenter prospective study by Brocco et al. (2022), who demonstrated higher <*n*> and <*D*_h_> of EPs in diluted blood from 56 patients with pancreatic cancer compared with 48 healthy individuals [39]. The use of FCM in this study employed fluorescence marking of EPs to quantify the subpopulations of EPs and relate the results to clinical outcomes [39]. However, the preparation of plasma and assessment of EPs in plasma remain of interest, as plasma is widely used in different fields of medicine for regeneration [81,82].

Although EPs in human IBD have previously been considered [83,84,85,86,87,88], to the best of our knowledge, we are the first to report on EPs in canine BOAS. As the interest in the application of EPs in veterinary medicine is expanding [89], it is expected that the study of animal samples will reveal important information about the general properties of EPs.

### 4.6. Challenges and Approaches

The International Society for Extracellular Vesicles (ISEV) is currently addressing the nomenclature, methods of harvesting, and the characterization of EPs, as well as conducting functional studies with various biological samples, to strengthen the approach to challenges related to EPs and to facilitate communication among scientists working in this multidisciplinary field. The ISEV recently published an update on the minimal information required for studies on extracellular vesicles [4], and the ISEV Blood EV Task Force created the Minimal Information for Blood EV Research (MIBlood-EV) [4], a tool to record and report information about pre-analytical protocols used for plasma and serum preparation, as well as the assays used to assess the quality of these preparations [90]. As all currently applied methods are subject to significant limitations, a combination of methods that exploit different physical and biochemical properties is recommended. However, the search for improved methods continues. ILM is a recently introduced method, which was hitherto used to detect viruses [26,27,91,92], phages [26,29], liposomes [28,93], and EPs [28,44,94,95]. Furthermore, interferometric NTA based on a laser light source offers the possibility of a better resolution down to smaller EP sizes [96]. Sausset et al. (2023) compared the performances of different techniques (ILM, TEM, and NTA) in the determination of the size of EPs isolated from bacteria, feces, bovine milk, and human cells, as well as phages of various sizes and shapes [29]. ILM performed well above its threshold 80 nm and it was suggested that ILM could be advantageous for large cohort studies, as the measurement is faster, simpler, and cheaper than NTA [29]. Our results are in favor of these suggestions; with our choice of protocols and equipment, we have assessed more than 700 samples in one year from patients included in the ongoing clinical studies, which is to our knowledge the largest number of EPs yet processed within a single design and setting.

In plasma and, particularly, in blood, there are also other particles besides EPs (e.g., lipoproteins, protein complexes, and cells). Although singular smaller particles are out of detection range of ILM, their group configuration could induce a fake signal, as previously observed for FCM [72,97]. In seeking the appropriate dilution of the samples, we observed that sometimes the signal was under the detection limit, but with increased dilution, the instrument was able to detect the particles, which aligns with FCM results detecting more particles with the dilution of samples [97]. The so-called swarm effect could explain the steep increase in the *D*_h_(*n*) curve with the decrease in *n* (Figure 1a–c). However, we observed on the camera display that ILM also detects signals moving synchronously with the cells. EPs could either be adhered to the cells or the cells exhibit protrusions of a size similar to the EPs. As cells are larger and less mobile than EPs, Brownian motion of the particle is hindered and *D*_h_ determined by the Stokes–Einstein relation overestimates the size of such particles. These artefacts were stronger in the blood than in the plasma, both in human and in the dog (Figure 1b and Figure 1c, respectively), and this could have contributed to larger <*D*_h_> in the blood than in the respective plasma (Table 2).

Although *D*_h_ and *n* are important measures to determine whether a sample preparation procedure affects EVs, they are insufficient to characterize whether that changes the EV internal content or membrane. Complementary techniques are needed to obtain information on such mechanisms regarding EPs and surrounding media (e.g., proteomic analysis [98,99,100,101]), as well as potential biological impacts. However, the complementary techniques should be subject to the same preparation of the sample.

## 5. Conclusions

To overcome the bottleneck to clinical relevance (formed by lengthy, sophisticated, deleterious, and poorly repeatable procedures that are invasive to samples), we analyzed populations of samples derived from clinical studies on two species (human and dog) for n and D_h_ by means of ILM. We experienced that the assessment of larger number of samples within populations by means of ILM is feasible. We found strong n and D_h_ correlations within and between human plasma and blood and between D_h_ in canine plasma and blood. We observed no statistically significant differences in the average number densities between the human samples. The value of <D_h_> was statistically significantly larger in blood than in plasma in human and in the dog. With ILM, improvements in the preparation of samples are needed, particularly for blood (e.g., personalized preparation of plasma and optimized dilution of plasma and of blood). To better understand the mechanisms taking place during processing of samples and to improve the methods, biophysical considerations of EPs will be highly warranted in the future.

## Figures and Tables

**Table 1:** Pearson correlation coefficients between *D*_h_ of EPs in human plasma and human blood; *n* of EPs in human plasma and human blood, collected in tubes with two different anticoagulants: trisodium citrate and EDTA, between *D*_h_ in plasma and blood, and between *n* in plasma and blood, in human and canine samples.

Human	*D*_h,citrate,plasma_	*D*_h,EDTA,plasma_	*D*_h,citrate,blood_	*D*_h,EDTA,blood_
*D*_h,citrate, plasma_	1	0.77 (p < 10^−5^, N = 151) *	0.48 (p < 10^−5^, N = 149) *	0.41 (p < 10^−5^, N = 146) *
*D*_h,EDTA, plasma_		1	0.39 (p < 10^−5^ N = 148) *	0.34 (p = 3 × 10^−5^, N = 145) *
*D*_h,citrate, blood_			1	0.61 (p < 10^−5^, N = 143) *
*D*_h,EDTA, blood_				1
	*n*_,citrate,plasma_	*n*_,EDTA,plasma_	*n*_,citrate,blood_	*n*_,EDTA,blood_
*n*_,citrate, plasma_	1	0.94 (p < 10^−5^, N = 150) *	0.49 (p < 10^−5^, N = 148) *	0.73 (p < 10^−5^, N = 146) *
*n*_,EDTA, plasma_		1	0.49 (p < 10^−5^, N = 146) *	0.81 (p < 10^−5^, N = 144) *
*n*_,citrate, blood_			1	0.47 (p < 10^−5^, N = 142) *
*n*_,EDTA, blood_				1
Human, Canine	*D*_h,human plasma_	*D*_h,canine plasma_	*n*_human plasma_	*n*_canine plasma_
*D*_h,human blood_	0.41 (p < 10^−5^, N = 294) *			
*D*_h,canine blood_		0.72 (p < 10^−5^, N = 76) *		
*n*_human blood_			0.66 (p < 10^−5^, N = 292) *	
*n*_canine blood_				0.07 (p = 0.55, N = 76)

Probability indicating the statistical significance of the correlation (p) and the number of samples included for each correlation (N) considered. Asterisks denote statistically significant correlations of *p* < 0.05.

**Table 2:** Average hydrodynamic diameter <*D*_h_> of EPs in human and canine plasma and in human and canine blood, the difference in the respective <*D*_h_>, and the corresponding probability of statistical significance (p) and statistical power (P) (in cases with p < 0.05). Human blood was collected in tubes with two different anticoagulants, trisodium citrate and EDTA; canine blood was collected in EDTA.

Sample	(<*D*_h,citrate_> ± SD) (nm)	(<*D*_h,EDTA_ > ± SD) (nm)	Difference (%)	p/P
Human plasma	140 ± 4 (N = 165)	137 ± 4 (N = 149)	−2	0.519
Human blood	155 ± 9 (N = 142)	162 ± 11 (N = 143)	4	0.645
Difference (%)	10	17		
p/P	<10^−5^/1 *	<10^−5^/1 *		
	(<*D*_h,plasma_> ± SD) (nm)	(<*D*_h,blood_> ± SD) (nm)		
Human	139 ± 31 (N = 315)	158 ± 11 (N = 285)	13	<10^−5^/1 *
Canine	155 ± 32 (N = 81)	171 ± 33 (N = 71)	10	0.003/0.82
Difference (%)	11	8		
p/P	<10^−5^/1 *	0.002/0.65		

The difference between any b and a was calculated as 2 × (b − a)/(a + b). N = the number of samples in the population. Asterisks mark statistically significant differences (p < 0.05) that have sufficient statistical power (P > 0.8).

**Table 3:** Average number density <*n*> of EPs in human and canine plasma and in human and canine blood, the difference in the respective <*n*>, the corresponding probability of statistical significance (p), and statistical power (P) (in cases with p < 0.05). Number densities are corrected for the dilution of samples. Human blood was collected in tubes with two different anticoagulants, trisodium citrate and EDTA; canine blood was collected in EDTA.

Sample	(<*n*_citrate_> ± SD) × 10^9^/mL	(<*n*_EDTA_> ± SD) × 10^9^/mL	Difference (%)	p/P
Human plasma	60.7 ± 65.2 (N = 165)	71.7 ± 152.5 (N = 149)	17	0.053
Human blood	63.2 ± 53.4 (N = 141)	58.8 ± 56.4 (N = 143)	−7	0.167
Difference (%) ^&^	4	−20		
p/P	0.759	0.239		
	(<*n*_plasma_> ± SD) × 10^9^/mL	(<*n*_blood_> ± SD) × 10^9^/mL		
Human	65.4 ± 154.9 (N = 314)	61.0 ± 49.4 (N = 285)	−7	0.461
Canine	46.8 ± 46.7 (N = 81)	73.7 ± 76.2 (N = 71)	45	0.009/0.8
Difference (%)	−33	22		
*p*/P	0.11	0.07		

^&^ The difference between a and b was calculated as 2 × (a − b)/(a + b). N = the number of samples in the population.

**Table 4:** Average measured (uncorrected for dilution) number density <*n*> of EPs in human plasma and in human blood collected in tubes with two different anticoagulants, trisodium citrate and EDTA and in canine plasma and canine blood, with the respective average dilution of the sample and number of particles measured (N_tracked_).

Sample	(<*n*_citrate_> ± SD) × 10^9^/mL	<Dilution_citrate_>	N_tracked_	(<*n*_EDTA_> ± SD) × 10^9^/mL	<Dilution_EDTA_>	N_tracked_
Human plasma	1.30 ± 0.84 (N = 163)	41 ± 53	114,772	1.31 ± 0.84 (N = 149)	46 ± 76	108,383
Human blood	0.22 ± 0.19 (N = 141)	302 ± 118	19,828	0.22 ± 0.28 (N = 142)	315 ± 116	18,375
Canine plasma				1.29 ± 1.18 (N = 81)	37 ± 25	48,998
Canine blood				0.30 ± 0.53 (N = 71)	348 ± 236	11,659

N = the number of samples in the population.

**Table 5:** Estimation of the size and number density of EPs in the selected studies.

Reference	Cohort	Method of Harvesting	Method of Assessment	N	Average Size (nm)	Average Number Density × 10^9^/mL
Blood						
This work	IBD	fresh, dilution	ILM	296	158 ± 11	61 ± 48
This work	canine BOAS	fresh, dilution	ILM	71	171 ± 33	75 ± 83
Marchisio et al., 2021 [37]	healthy	fresh, dilution	FCM	22	>160 nm	0.0083 ± 0.0042
Brocco et al., 2022 [38]	pancreatic cancer	fresh, dilution	FCM	56		0.0021
Brocco et al., 2022 [38]	healthy	fresh, dilution	FCM	48		0.0016
Plasma						
This work	IBD	fresh, dilution	ILM	303	136 ± 31	68 ± 106
This work	canine BOAS	fresh, dilution	ILM	81	155 ± 32	49 ± 48
Berry et al., 2024 [44]	multispecies	fresh, dilution	ILM	250	130–200	
Kogej et al., 2021 [23]	ovarian cancer	fresh, dilution	DLS	7	20–40	
Mork et al., 2016 [41]	healthy	fresh, dilution	NTA	20	42–73	89–1000
Mork et al., 2016 [41]	healthy	fresh, dilution	TRPS	20	171–276	0.21–1.30
de Vrij et al., 2013 [42]	healthy	fresh, dilution	TRPS	NS	≈200	0.2
Gardiner et al., 2013 [35]	NS	NS	NTA	NS		1000–5000
Holcar et al., 2024 [52]	healthy	thawed	FCM	208		0.0002–0.042
Botha et al., 2021 [43]	NS	thawed	FCM	NS		<≈0.0008
Robinson et al., 2024 [40]	healthy	thawed	NTA	3	≈(65–80)	987
Lawrie et al., 2009 [20]	healthy	thawed	DLS	20	77–266	
Isolated EVs						
Gardiner et al.,2013 [35]	NS	UC	NTA	NS	70–120	5–50
Elgamal et al., 2021 [53]	NS	UC	NTA	44		219 ± 9.5
Osti et al., 2019 [54]	glioblastoma preop.	UC	NTA	13	95 ± 20	69.7 ± 9.7
Osti et al., 2019 [54]	healthy	UC	NTA	17	100 ± 15	21.2 ± 2.8
Osti et al., 2019 [54]	glioblastoma postop.	UC	NTA	30	120 ± 12	34.5 ± 3.8
Osti et al., 2019 [54]	healthy	UC	NTA	16	128 ± 20	19.6 ± 2.2
Bettio et al., 2023 [55]	healthy	UC	NTA	30	153	17.8
Jamaly et al., 2018 [56]	healthy	UC	NTA	10	≈(70–90)	≈(20–25)
Serrano-Pertierra et al., 2019 [57]	healthy	UC	DLS	3	152.09 ± 29.38	
Serrano-Pertierra et al., 2019 [57]	healthy	UC	NTA	3	208.5 ± 10.60	603
Malys et al., 2021 [58]	healthy	UC	NTA	9	148.11 ± 14.64	0.27 ± 0.22
George et al., 2021 [59]	NS	UC	NTA	NS	150	400 ± 170
Holcar et al., 2024 [52]	healthy	UC	NTA	208	152	5.7
Scavo et al., 2019 [60]	healthy	UC	DLS	8	132	
Scavo et al., 2019 [60]	colorectal cancer	UC	DLS	22	151	
Scavo et al., 2019 [60]	gastric cancer	UC	DLS	8	157	
Dlugolecka et al., 2021 [61]	lung cancer	UC	NTA	34	98.43 ± 10.31	244 ± 471
Picciolini et al., 2021 [62]	healthy	SEC	NTA	10	167 ± 9.4	23.4
Picciolini et al.,2021 [62]	Alzheimer’s disease	SEC	NTA	10	183 ± 4.6	118
Contreras et al., 2023 [63]	healthy	SEC	NTA	3	119.4 ± 6.9	
Stranska et al., 2018 [64]	healthy	SEC	NTA	6	117	10
Diehl et al., 2023 [65]	healthy	SEC	TRPS	12	77.75	60
Diehl et al., 2023 [65]	Marfan syndrome	SEC	TRPS	6	69.92	147
Franco et al., 2023 [66]	healthy, idiopathic inflammatory myopathy	SEC, UF	NTA	70	202 ± 19	15.1 ± 10.6
Malys et al., 2021 [58]	healthy	SEC	NTA	9	132.52 ± 12.05	4975.6 ± 769.5
Serrano-Pertierra et al., 2019 [57]	healthy	precipitation	DLS	3	76.64 ± 16.17	
Serrano-Pertierra et al., 2019 [57]	healthy	precipitation	NTA	3	203.67 ± 55.41	1263
Longobardi et al., 2021 [67]	Alzheimer’s	precipitation	NTA	30	133.8 ± 17.1	147 ± 61.8
Longobardi et al., 2021 [67]	frontotemporal dementia	precipitation	NTA	30	130.2 ± 18.8	139 ± 61
Longobardi et al., 2021 [67]	dementia with Lewy bodies	precipitation	NTA	30	127.2 ± 12.0	126 ± 51
Longobardi et al., 2021 [67]	healthy	precipitation	NTA	30	113 ± 13.2	249 ± 118
Serrano-Pertierra et al., 2019 [57]	healthy	precipitation	DLS	3	123.55 ± 63.04	
Serrano-Pertierra et al., 2019 [57]	healthy	precipitation	NTA	3	233.97 ± 33.73	7383
König et al., 2017 [68]	breast cancer	precipitation	NTA	105	140	≈2500
König et al., 2017 [68]	healthy	precipitation	NTA	16		≈60
Giloteux et al., 2020 [69]	myalgic encephalomyelitis/chronic fatigue syndrome	precipitation	NTA	35	130.1 ± 12.7	7.6 ± 5.0
Giloteux et al., 2020 [69]	healthy	precipitation	NTA	35	132.7 ± 16.4	6.6 ± 4.3
Stranska et al., 2018 [64]	healthy	membrane-based affinity binding	NTA	6	210	40
Bettio et al., 2023 [55]	healthy	membrane-based affinity binding	NTA	30	238	23.2
Scavo et al., 2019 [60]	healthy	UC	TEM	8	87	
Scavo et al., 2019 [60]	colorectal cancer	UC	TEM	22	108	
Scavo et al., 2019 [60]	gastric cancer	UC	TEM	8	112	

N: number of cases; NS: nonspecified; UF: ultrafiltration.

## Data Availability

The raw data are provided in the Supplementary Materials.

## Supplementary Materials

The following supporting information can be downloaded at: https://www.mdpi.com/article/10.3390/cells13242054/s1, The raw data are available in Korenjak, B., Tratenšek, A., Arko, M., Romolo, A., Berry, M., Drobne, D., Vovk, T., Iglič, A., Nemec Svete, A., Erjavec, V., & Kralj-Iglič, V. (2024). Raw Data on Extracellular Particles in 613 Human and 163 Canine Diluted Plasma and Blood Samples Assessed by Interferometric Light Microscopy (1.0.0) [Data set]. Zenodo. https://doi.org/10.5281/zenodo.14015248.
