# Supplementary material for: Assessment of Extracellular Particles Directly in Diluted Plasma and Blood by Interferometric Light Microscopy. A Study of 613 Human and 163 Canine Samples
Source: Cells. 2024 Dec 12;13(24):2054. doi: 10.3390/cells13242054 (PMC11674815; doi:10.3390/cells13242054)
Supplement: Supplementary file 1 [file cells-13-02054-s001.zip › cells-3263040-supplementary.pdf]

Raw data on extracellular particles in human plasma and blood, and canine plasma and blood

Legend
S: sample
Dil.: dilution
n: number density of EPs
corr *n*: number density of EPs corrected for dilution
D_h: hydrodynamic diameter of EPs
W: width of the EPs distribution over D_h
$N_{tracked}$: number of particles tracked

Human plasma trisodium citrate						
S	Dil.	$n \times 10^9$/mL	corr $n \times 10^9$/mL	D_h(nm)	W(nm)	$N_{tracked}$
H1	50	1.05	52.33	131.67	73	693
H2	50	1.06	53.00	115.67	78	722
H3	25	0.40	10.08	152.67	105	236
H4	50	2.30	115.00	153.67	68	1015
H5	50	1.23	61.50	122.67	73	262
H6	20	0.63	12.60	146.00	146	410
H7	50	0.69	34.67	128.67	75	396
H8	50	1.61	80.50	137.00	78	945
H9	50	0.99	49.67	117.00	43	615
H10	50	0.88	43.83	136.00	71	678
H11	50	3.03	151.67	127.67	94	1056
H12	50	3.02	151.17	131.00	60	1022
H13	10	0.51	5.13	208.67	116	388
H14	50	0.45	22.67	126.00	81	291
H15	50	1.18	59.17	130.67	64	898
H16	20	1.37	27.47	124.67	71	891
H17	50	0.43	21.67	135.00	78	361
H18	2	1.05	2.09	196.33	117	897
H19	20	0.61	12.13	125.33	63	478
H20	10	0.52	5.23	163.33	103	394
H21	50	2.28	114.17	146.33	85	943
H22	50	0.93	46.67	129.00	65	633
H23	50	0.81	40.50	119.33	56	430
H24	50	1.70	85.00	128.33	50	933
H25	50	1.95	97.33	134.33	53	990
H26	25	0.51	12.83	126.00	77	269
H27	20	0.67	13.33	153.33	181	492
H28	50	0.59	29.67	154.67	109	302
H29	25	0.55	13.83	145.33	98	558
H30	20	0.67	13.47	144.00	132	566
H31	20	1.22	24.40	127.67	98	863
H32	20	0.74	14.80	214.67	142	476
H33	10	1.41	14.10	151.00	86	954
H34	20	0.66	13.27	161.33	161	409
H35	10	1.01	10.13	127.67	65	745
H36	200	2.23	445.33	134.00	95	1020
H37	50	3.84	192.17	127.00	54	1096
H38	20	1.39	27.80	137.00	85	907
H39	50	0.48	24.00	178.33	177	406
H40	50	0.39	19.50	167.00	169	62
H41	50	3.07	153.33	141.67	66	995
H42	10	0.63	6.30	138.00	132	598
H43	50	0.65	32.33	141.67	94	632
H44	50	0.62	30.83	131.67	85	483
H45	50	1.75	87.50	126.33	66	949
H46	50	1.04	52.00	120.67	68	786
H47	20	0.84	16.80	130.33	67	651
H48	50	0.60	30.00	146.67	71	374
H49	20	1.20	24.07	132.33	56	832
H50	50	0.88	44.00	129.33	60	649
H51	50	2.99	149.67	133.00	71	951
H52	50	0.50	24.83	126.67	55	390
H53	50	1.23	61.67	125.00	71	900
H54	50	0.80	39.83	130.67	70	733
H55	50	2.99	149.33	135.00	62	982
H56	50	1.56	78.17	133.00	83	956
H57	50	0.56	28.00	130.00	68	456
H58	20	0.77	15.33	145.00	134	636
H59	20	1.92	38.33	156.33	81	838
H60	50	1.10	54.83	126.00	62	765
H61	50	1.90	94.83	124.00	44	979

Human plasma EDTA						
S	Dil.	$n \times 10^9$/mL	corr $n \times 10^9$/mL	D_h(nm)	W(nm)	$N_{tracked}$
H1	50	1.44	72.00	132.00	58	938
H2	50	1.30	65.00	121.00	50	810
H3	25	0.35	8.67	145.67	115	191
H4	50	1.88	94.00	137.67	67	954
H5	50	2.84	141.83	122.00	51	1012
H6	10	0.60	6.00	141.67	82	506
H7	50	0.66	33.00	127.00	60	421
H8	50	1.56	77.83	139.00	77	978
H9	50	1.23	61.67	122.67	51	808
H10	5	1.35	6.75	157.00	105	990
H11	50	3.31	165.33	128.67	57	992
H12	50	3.40	169.83	140.33	54	1013
H13	5	0.52	2.58	218.00	127	394
H14	50	0.49	24.67	141.67	92	310
H15	50	1.45	72.67	132.33	57	961
H16	50	0.88	44.17	117.67	56	648
H17	50	0.37	18.50	124.33	68	293
H18	5	0.95	4.73	252.00	136	908
H19	20	0.74	14.80	131.00	76	578
H20	10	0.44	4.43	154.67	107	354
H21	50	1.56	77.83	126.33	48	984
H22	50	1.02	50.83	133.33	53	689
H23	50	0.86	43.17	123.33	81	477
H24	50	2.16	53.92	129.00	53	989
H25	50	1.51	75.50	131.00	59	962
H26	25	0.50	12.42	134.33	76	253
H27	10	0.75	7.50	153.33	96	665
H28	10	0.74	7.40	144.67	72	709
H29	25	0.53	13.33	141.33	83	422
H30	20	0.67	13.47	139.00	84	612
H31	20	1.16	23.20	126.00	68	863
H32	24	0.26	6.16	169.67	105	181
H33	10	1.39	13.87	150.67	84	940
H34	10	0.59	5.87	147.00	121	453
H35	20	0.78	15.60	127.00	69	619
H36	100	3.58	357.67	138.67	72	945
H37	200	2.51	502.00	119.67	54	948
H38	50	0.83	41.67	129.00	62	705
H39	10	0.74	7.43	170.67	120	619
H40	50	0.61	30.33	149.00	71	375
H41	50	3.80	189.83	136.67	62	953
H42	10	0.61	6.10	142.67	84	601
H43	50	2.15	107.50	118.00	46	973
H44	50	0.95	47.67	126.33	52	696
H45	50			126.33	71	935
H46	50	1.13	56.50	126.67	54	805
H47	20	0.71	14.13	138.67	81	588
H48	10	0.33	3.30	151.00	129	187
H49	50	0.59	29.67	126.67	55	489
H50	50	0.99	49.67	129.00	55	769
H51	50	3.29	164.33	131.67	58	1077
H52	20	0.88	17.67	131.00	65	692
H53	50	1.19	59.33	128.67	60	822
H54	50	0.72	36.17	131.00	53	608
H55	50	2.74	136.75	148.50	67	699
H56	50	1.45	72.33	130.00	58	954
H57	20	1.07	21.40	145.00	109	842
H58	20	0.92	18.33	141.33	90	703
H59	10	0.33	3.30	219.00	117	55
H60	50	0.76	37.83	128.33	62	630
H61	50	2.46	123.17	126.33	47	970

H62	50	2.16	108.00	122.67	49	998
H63	50	2.56	127.83	122.00	54	1006
H64	50	0.66	33.00	138.33	76	404
H65	50	1.26	63.17	125.67	71	818
H66	50	1.89	94.33	138.33	71	990
H67	20	0.60	11.93	165.00	115	432
H68	50	0.50	24.83	133.00	116	362
H69	50	0.70	35.17	124.33	69	565
H70	10	1.35	13.50	127.00	75	898
H71	10	1.94	19.43	143.00	76	929
H72	50	2.66	133.17	125.67	61	944
H73	10	0.61	6.07	147.00	117	516
H74	50	0.63	31.33	139.67	116	481
H75	50	0.87	43.67	129.67	71	659
H76	100	3.73	373.00	120.67	44	944
H77	50	1.09	54.50	131.33	61	695
H78	500	0.74	371.67	121.00	76	594
H79	20	0.93	18.53	121.00	90	693
H80	20	1.11	22.13	131.67	59	854
H81	20	1.01	20.13	146.67	96	791
H82	50	1.68	84.17	129.33	87	997
H83	20	3.12	62.33	136.33	61	1034
H84	20	0.88	17.53	143.67	79	712
H85	20	1.45	29.07	157.33	91	895
H86	50	0.76	37.83	123.00	79	579
H87	10	0.64	6.37	137.33	138	523
H88	50	1.03	51.50	130.00	60	760
H89	50	0.89	44.33	128.33	85	825
H90	200	3.42	684.67	135.33	70	1113
H91	50	2.81	140.50	129.00	69	1070
H92	50	1.16	58.17	130.33	66	784
H93	50	3.22	160.83	125.67	56	984
H94	50	1.21	60.67	132.33	59	889
H95	50	0.57	28.33	132.33	57	495
H96	20	0.77	15.33	145.00	134	636
H97	5	0.86	4.28	151.67	88	763
H98	50	1.84	92.00	130.00	57	992
H99	10		8.77	151.33	**130**	373
H100	20		7.13	125.33	**121**	278
H101	20	1.60	32.00	155.00	101	1013
H102	50	2.71	135.50	136.00	83	1057
H103	50	0.84	41.83	119.33	104	614
H104	50	2.97	148.67	144.67	106	1039
H105	50	0.56	27.83	128.67	112	400
H106	50	0.95	47.67	133.00	79	745
H107	50	0.77	38.33	134.00	139	614
H108	20	0.80	15.93	200.67	123	660
H109	50	1.21	60.50	126.33	90	828
H110	20	0.53	10.67	151.00	167	446
H111	50	2.41	120.50	136.67	67	969
H112	20	1.86	37.20	184.67	100	952
H113	20	0.87	17.33	116.00	109	593
H114	20	0.62	12.33	131.33	70	480
H115	20	1.35	27.07	128.00	112	939
H116	50	2.83	141.50	122.00	49	984
H117	5	1.02	5.08	162.67	140	791
H118	50	2.49	124.33	133.00	53	1020
H119	20	0.65	13.07	153.00	102	604
H120	5	1.03	5.13	145.33	127	919
H121	200	1.94	388.67	132.33	78	1007
H122	50	0.79	39.33	126.33	76	595
H123	20	1.01	20.13	125.33	71	720
H124	5	0.64	3.22	175.67	146	523
H125	50	0.86	42.83	125.67	64	661
H126	50	2.29	114.67	126.00	50	1003
H127	50	2.06	103.17	125.33	65	950
H128	50	1.11	55.67	127.00	79	772
H129	5	0.62	3.08	177.33	183	570
H130	5	0.81	4.05	153.00	125	757
H131	10	1.01	10.10	134.33	82	731
H132	50	0.68	33.83	120.00	80	538
H133	5	0.69	3.45	147.33	174	630
H134	50	0.76	37.83	124.00	50	606
H135	50	0.94	47.00	143.67	73	733

H62	50	2.15	107.67	120.67	45	936
H63	50	2.66	133.17	125.67	54	996
H64	50	0.60	30.00	126.00	80	358
H65	50	1.71	85.67	125.33	41	936
H66	50	2.10	104.83	137.67	61	934
H67	50	0.24	12.00	121.00		26
H68	50	0.74	36.83	138.67	113	626
H69	50	0.57	28.33	123.33	54	560
H70	10	1.39	13.87	125.00	53	909
H71	10	1.88	18.77	140.33	76	974
H72	50	3.04	152.17	125.00	50	1027
H73	10	0.51	5.13	152.33	121	476
H74	50	0.59	29.33	132.33	57	444
H75	50	1.12	55.83	127.00	53	813
H76	100	3.87	386.67	119.67	37	1064
H77	50	1.15	57.33	125.33	65	816
H78	500	1.21	605.00	119.67	62	830
H79	20	1.03	20.60	124.67	64	766
H80	20	1.00	20.00	134.33	70	793
H81	50	0.58	29.00	137.67	78	487
H82	50	1.94	97.00	124.00	61	1012
H83	10	0.53	5.30	158.33	102	445
H84	20	0.73	14.53	141.33	52	642
H85	50	1.01	50.25	143.00	70	470
H86	20	1.25	25.00	126.33	72	858
H87	10	0.60	6.00	141.33	103	500
H88	50	0.97	48.50	130.67	60	734
H89	50	0.86	43.00	124.33	76	683
H90	500	2.18	1088.33	127.00	52	1037
H91	50	3.12	155.83	127.00	57	1037
H92	50	1.17	58.67	130.00	84	938
H93	50	3.37	168.33	131.33	54	1040
H94	50	1.25	62.50	141.67	54	830
H95	50	0.79	39.50	143.67	68	838
H96	20	0.92	18.33	141.33	90	703
H97	5	0.67	3.35	181.00	117	636
H98	50	2.20	110.00	129.33	48	982
H99	10				102	366
H100	10				70	939
H101	20	1.18	23.60	137.33	85	868
H102	50	2.45	122.50	138.33	62	945
H103	50	0.74	36.83	120.00	75	529
H104	100	2.21	221.33	135.33	59	1002
H105	20	1.43	28.53	136.33	76	969
H106	50	0.88	43.83	129.00	77	717
H107	10	0.67	6.70	137.67	80	647
H108						
H109	50	1.30	65.17	122.33	46	951
H110	20	1.60	32.00	193.00	87	940
H111	50	2.45	122.50	128.00	68	1005
H112	20	1.01	20.13	142.67	107	773
H113	20	0.97	19.33	119.67	78	685
H114	20	0.66	13.13	129.33	72	418
H115	20	1.57	31.40	129.67	54	982
H116	100	2.24	223.67	120.00	45	947
H117	5	0.92	4.58	156.67	76	776
H118	50	2.34	117.00	126.67	49	948
H119	20	0.79	15.87	151.00	101	669
H120	20	0.66	13.20	138.67	89	492
H121	200	2.16	432.67	127.67	58	974
H122	50	0.70	34.83	131.00	56	578
H123	20	1.18	23.67	130.00	89	853
H124	5	0.95	4.75	233.00	106	790
H125	50	0.77	38.67	127.67	57	654
H126	50	2.79	139.67	126.00	49	1029
H127	50	2.20	109.83	121.67	48	919
H128	50	1.02	51.00	128.67	48	755
H129	5	0.67	3.37	161.33	115	632
H130	5	0.80	4.00	155.00	99	757
H131	10	0.94	9.40	131.33	65	754
H132	50	0.55	27.50	127.33	73	478
H133	5	0.63	3.15	150.00	141	588
H134	50	0.98	49.17	128.00	53	743
H135	50	1.16	58.17	136.33	58	806

H136	50	0.70	35.17	126.33	61	565
H137	50	0.60	29.83	125.33	57	508
H138	50	1.26	63.17	132.00	78	885
H139	50	2.06	102.83	196.33	124	818
H140	50	0.73	36.67	120.00	42	590
H141	2	1.43	2.87	201.67	98	960
H142	20	1.74	34.80	164.33	97	992
H143	20	0.54	10.73	153.67	126	434
H144	50	1.48	74.17	128.33	56	967
H145	20	0.92	18.47	134.33	64	693
H146	50	2.33	116.67	129.00	78	995
H147	50	2.92	145.83	122.33	68	994
H148	50	0.76	37.83	129.33	51	612
H149	20	0.68	13.53	149.00	101	570
H150	50	2.04	102.17	129.00	62	671
H151	50	1.64	82.17	119.33	44	966
H152	2	0.64	1.28	253.33	154	655
H153	20	0.90	18.00	143.33	83	721
H154	20	0.53	10.60	249.67	129	427
H155	10	0.58	5.83	206.67	115	532
H156	5	0.62	3.10	228.00	91	337
H157	10	0.35	3.53	180.67	144	337
H158	20	0.36	7.27	117.00	86	462
H159	20	3.75	75.00	125.00	72	1025
H160	50	2.36	117.83	132.33	68	980
H161	50	2.24	112.00	124.67	76	1021
H162	50	2.45	122.67	125.00	60	989
H163	10	1.96	19.63	127.00	78	936
H164	10	0.37	3.67	119.67	94	504
H165	20	0.50	10.07	115.33	90	590
H166	5	0.32	1.58	174.67	131	496

H136	50	1.00	49.83	119.67	50	723
H137	50	0.61	30.33	120.67	43	467
H138	50	1.28	64.17	133.00	66	882
H139	50	0.56	28.17	128.67	84	403
H140	50	0.99	49.50	120.33	55	712
H141	4	2.45	9.79	246.33	109	1055
H142	20	1.29	25.87	149.00	95	947
H143	20	0.53	10.60	168.67	140	432
H144	50	1.53	76.50	128.33	49	941
H145	20	0.87	17.33	130.33	57	696
H146	50	2.29	114.50	127.33	60	1012
H147	50	2.33	116.33	121.67	57	1010
H148	50	1.00	50.00	131.67	79	776
H149	20	0.88	17.67	144.00	123	737
H150	50	2.51	125.67	129.33	48	1088
H151	50	1.56	78.00	114.67	44	909
H152	2	0.66	1.33	288.67	171	687
H153	20	0.90	18.07	147.67	84	786
H154	10	1.08	10.80	167.67	98	763

Human blood trisodium citrate						
S	Dil.	$n \times 10^9$/mL	corr $n \times 10^9$/mL	D_h(nm)	W(nm)	$N_{tracked}$
H1	400	0.13	53.33	137.33	128	69
H2	400	0.15	60.00	120.33	68	78
H3	400	0.34	134.67	174.33	74	170
H4	400	0.20	78.67	163.00	99	59
H5	400	0.13	52.00	128.67		46
H6	800	0.10	77.33	380.00		46
H7	400	0.21	82.67	169.00	50	90
H8	400	0.14	57.33	160.67		73
H9	400	0.15	60.00	143.00	45	68
H10	200	0.14	28.67	167.33	99	72
H11	400	0.29	114.67	129.33	75	172
H12	400	0.28	112.00	121.33	46	163
H13	200	0.11	22.00	156.00		19
H14	400	0.12	46.67	120.67		35
H15	400	0.18	73.33	144.33	71	120
H16	400	0.15	61.33	110.67	37	83
H17	800	0.13	104.00	233.00	307	52
H18	200	0.14	27.00	194.67	83	68
H19	200	0.13	26.00	203.00	44	41
H20	200	0.12	24.67	156.67		54
H21	400			204.33	99	999
H22	400	0.14	57.33	183.33	149	76
H23	400	0.80	321.33	120.67	74	457
H24	400	0.24	97.33	131.67	66	150
H25	400	0.28	110.67	146.00	56	221
H26	400	0.13	53.33	202.67		54
H27	400	0.21	82.67	148.33	97	106
H28	400	0.27	109.33	184.67	84	
H29	400	0.23	93.33	168.67	101	193
H30	400	0.12	46.67	170.00		40
H31	400	0.20	78.67	145.67	114	105
H32	400	0.18	72.00	211.33	45	75
H33	400	0.63	250.67	146.00	108	561
H34						
H35	200	0.12	23.33	145.33		243
H36	200	0.84	168.00	145.67	72	817
H37	200	0.92	184.00	131.67	52	877

Human blood EDTA						
S	Dil.	$n \times 10^9$/mL	corr $n \times 10^9$/mL	D_h(nm)	W(nm)	$N_{tracked}$
H1	400	0.12	46.67	128.33		37
H2	400	0.19	76.00	117.00	53	105
H3	400	0.11	44.00	179.00		28
H4	600	0.13	80.00	121.00		59
H5	400	0.18	72.00	188.00		26
H6	600	0.17	100.00	215.00	111	81
H7	400	0.10	41.33	139.67		45
H8	400	0.15	58.67	163.33		73
H9	600	0.14	84.00	149.33		58
H10	200	0.16	32.00	145.00		81
H11	400	0.40	158.67	119.67	68	234
H12	400	0.31	124.00	122.00	49	189
H13	400	0.11	45.33	153.33		37
H14	400	0.12	49.33	208.33		37
H15	400	0.16	64.00	133.00		74
H16	400	0.13	53.33	119.00		55
H17	400	0.07	26.67	147.00		19
H18	200	0.10	20.00	210.67		40
H19	200	0.15	30.00	180.33		64
H20	400	0.10	41.33	199.33		25
H21	400	0.15	58.67	152.33	90	67
H22	400	0.12	48.00	125.67		43
H23	400	0.12	48.00	128.33		37
H24	400	0.24	94.67	132.67	83	133
H25	400	0.25	100.00	139.67	77	233
H26	600	0.12	74.00	226.33		42
H27	400	0.18	72.00	167.00	85	92
H28	400	0.13	53.33	235.33	140	72
H29	400	0.12	46.67	253.67	80	64
H30	400	0.14	54.67	204.00		61
H31	400	0.18	70.67	134.67	70	89
H32	400	0.21	82.67	175.00	117	124
H33	400	0.11	45.33	176.00		52
H34	10	0.49	4.93	150.67	163	568
H35	600	0.10	62.00	190.67		40
H36	200	0.91	182.00	145.67	80	919
H37	200	0.92	183.33	126.33	61	918

H38	400	0.17	68.00	159.33	141	92
H39	200	0.17	33.33	205.67	107	99
H40						
H41	100	0.61	61.33	149.33	108	665
H42	200	0.38	75.33	196.33	134	535
H43	200	0.28	56.67	126.67	72	255
H44	200	0.16	32.00	137.33	166	76
H45	200	0.30	59.33	130.00	45	243
H46	200	0.19	37.33	148.33	74	125
H47	400	0.11	44.00	149.00		28
H48	200	0.14	27.00	124.50		22
H49	400	0.12	46.67	156.33		46
H50	200	0.15	30.67	160.67	57	105
H51	200	0.45	90.00	132.67	60	462
H52	200	0.12	24.00	126.67		37
H53	200	0.21	41.33	145.67	84	80
H54	400	0.16	65.33	141.67	54	77
H55	200	0.45	90.00	142.00	59	431
H56	200	0.31	62.00	149.00	124	260
H57	200	0.22	44.00	140.67	78	198
H58	200	0.40	80.67	156.67	81	303
H59	400	0.40	161.33	176.00		24
H60	200	0.19	37.33	132.67	72	130
H61	200	0.25	49.33	123.00	60	222
H62	200	0.26	52.00	130.67	50	235
H63	200	0.34	67.33	131.00	83	301
H64	200	0.12	24.00	213.33	115	88
H65	200	0.22	43.33	136.00	56	132
H66	200	0.31	62.00	140.67	92	237
H67	400	0.11	45.33	204.67		22
H68	200	0.21	42.67	147.33	68	152
H69	200	0.15	30.00	179.00	94	123
H70	400	0.12	46.67	202.67		41
H71	200	0.14	28.00	141.33	44	87
H72	200	0.26	51.33	133.00	78	149
H73	400	0.12	49.33	195.33		38
H74	200	0.17	33.33	152.33	64	82
H75	200					
H76	400	0.34	137.33	124.00	78	268
H77	400	0.13	53.33	135.00	65	66
H78	400	0.59	236.00	131.00	60	496
H79	600	0.12	72.00	158.33		44
H80	200	0.33	65.33	204.33	115	260
H81	200	0.13	26.00	151.00		58
H82	200	0.35	69.33	124.67	83	286
H83	200	0.20	39.33	177.67	111	108
H84	400	0.14	57.33	223.33	136	70
H85	400	0.13	50.67	169.00		46
H86	400	0.15	60.00	145.00	59	61
H87	400	0.15	58.67	193.33	201	76
H88	200	0.13	25.33	143.00	59	72
H89	200	0.15	29.33	148.67	103	105
H90	200	0.96	192.67	129.33	54	746
H91	200	0.41	82.67	134.67	67	421
H92	400	0.15	61.33	138.00		83
H93	200	0.58	116.67	126.67	50	556
H94	200	0.22	44.00	138.67	63	216
H95	200	0.20	39.33	136.33	62	111
H96	200	0.40	80.67	156.67	81	303
H97	200	0.15	29.33	163.67	54	62
H98	200	0.24	47.33	144.33	125	192
H99						
H100						
H101	400	0.12	46.67	151.67		30
H102	200	0.22	44.67	151.33	62	200
H103	400	0.13	52.00	138.67		48
H104	200	0.31	62.00	142.00	61	289
H105	200	0.12	24.00	175.33		41
H106	400	0.13	53.33	171.00	85	67
H107	400	0.11	45.33	191.33		27
H108	200	0.15	29.33	259.33	105	68
H109	200	0.18	35.33	129.67	46	98
H110	400	0.11	42.67	170.33		31
H111	200	0.44	88.00	140.00	67	365

H38	400	0.12	46.67	151.67	67	53
H39	200	0.25	50.67	216.33	99	235
H40	200	0.30	60.67	225.33	92	315
H41	100	0.69	69.33	144.67	62	745
H42	200	0.62	123.33	140.67	75	583
H43	400	0.25	98.67	130.33	70	175
H44	200	0.15	30.00	152.67	78	87
H45	200	0.34	67.33	141.67	82	247
H46	200	0.15	30.67	137.33	86	83
H47	400	0.15	60.00	149.00		46
H48	200	0.16	31.33	188.33		104
H49	400					
H50	200	0.13	26.67	148.33	170	90
H51	200	0.49	97.33	135.67	61	444
H52	400	0.14	56.00	143.00		54
H53	400	0.24	96.00	159.50	80	145
H54	200	0.18	35.33	140.67	58	114
H55	200	0.49	97.33	139.67	56	476
H56	200	0.27	54.67	127.67	74	265
H57	200	0.13	26.00	141.33		49
H58	400	0.15	60.00	184.33	112	86
H59	600	0.11	64.00	178.00		28
H60	200	0.16	32.67	129.67	55	126
H61	200	0.25	50.67	123.00	44	247
H62	200	0.25	49.33	118.00	49	193
H63	200	0.25	49.33	147.00	56	172
H64	200	0.17	34.67	197.33	75	148
H65	200	0.21	42.00	123.33	42	128
H66	200	0.18	35.33	132.00	91	132
H67	200	0.36	72.00	155.67	77	334
H68	200	0.13	26.00	157.00	91	59
H69	200	0.15	29.33	140.33	93	109
H70	400	0.13	52.00	190.67		37
H71	200	0.13	26.67	150.00	176	76
H72	200	0.27	54.00	129.00	58	250
H73						
H74	200	0.16	32.00	112.00	56	91
H75	200	0.12	24.00	129.50		26
H76	400	0.39	154.67	127.67	51	298
H77	400	0.24	94.67	209.67	176	182
H78						
H79	200	0.40	79.33	210.00	73	353
H80	200	0.27	54.00	206.67	94	221
H81	800	0.14	114.67	239.00	257	125
H82	200	0.25	50.67	126.33	49	229
H83	10	0.38	3.80	171.67	94	211
H84	600	0.12	72.00	192.33		53
H85						
H86	400	0.12	49.33	154.33	216	60
H87	200	0.16	32.67	214.67	133	102
H88	200	0.12	24.00	158.33	118	82
H89	400	0.13	52.00	144.00	61	82
H90	200	2.27	454.67	134.67	82	932
H91	200	0.53	105.33	126.33	57	447
H92	200	0.23	46.67	141.67	63	263
H93	200	0.43	86.00	135.00	62	493
H94	200	0.21	42.00	149.00	62	154
H95	200	0.19	37.33	128.67	67	122
H96	400	0.15	60.00	184.33	112	86
H97						
H98	200	0.24	48.67	133.00	81	191
H99						
H100						
H101	400	0.10	40.00	180.00		16
H102	200	0.17	33.33	152.67	65	183
H103	600	0.15	88.00	139.67		66
H104	200	0.25	50.00	143.00	61	276
H105	400	0.13	52.00	212.33		50
H106	400	0.14	57.33	176.33	83	60
H107	200	0.13	25.33	229.67	211	124
H108	600	0.23	135.00	200.50		59
H109	200	0.18	35.33	127.67	46	96
H110	200	0.11	21.33	164.00		29
H111	200	0.31	62.00	130.33	99	282

H112	200	0.13	25.33	151.67	132	45
H113	600	0.11	64.00	223.33		23
H114	400	0.12	48.00	191.33		24
H115	200	0.15	30.00	149.67	54	70
H116	200	0.32	64.00	129.33	64	293
H117	200	0.12	24.00	277.33		37
H118	200	0.24	48.67	138.33	50	211
H119	400	0.11	44.00	160.67		31
H120	200	0.12	23.33	196.67		42
H121	200	0.62	124.00	144.67	75	42
H122	200	0.14	27.33	128.00		55
H123	200	0.13	26.00	144.67		59
H124	200	0.12	24.00	206.00		41
H125	200	0.14	28.67	136.67		68
H126	200	0.24	48.00	132.00	56	168
H127	200	0.27	54.67	122.67	40	186
H128	200	0.18	36.00	146.67	120	103
H129	400	0.10	41.33	199.67		17
H130	400	0.12	46.67	198.33		25
H131	400	0.11	45.33	168.33		20
H132	200	0.13	25.33	175.00	90	69
H133	200	0.11	22.67	240.33		32
H134	200	0.12	24.67	118.67	105	57
H135	200	0.14	27.33	162.33	45	78
H136	400	0.16	62.67	127.00		59
H137	600	0.18	110.00	146.00	132	113
H138	200	0.14	28.00	137.33	71	89
H139	600	0.26	158.00	214.33	107	153
H140	400	0.13	53.33	121.00		45
H141	400	0.13	50.67	369.67		46
H142	400	0.10	41.87	129.33		24
H143	600	0.11	66.00	251.33		39
H144	200	0.23	45.33	132.00	53	155
H145	400	0.12	48.00	263.33		55
H146	200	0.26	52.67	131.67	67	243
H147	200	0.18	35.33	130.00	70	178
H148	200	0.14	27.33	153.67		59
H149	800	0.14	109.33	133.67		61
H150	200	0.19	38.00	153.00	62	169
H151	200	0.13	26.00	134.67	84	86
H152	800	0.12	93.33	167.67		31
H153	400	0.13	50.67	151.00		44
H154	400		44.00	254.67		30

H112	400	0.11	45.33	212.00		37
H113	600	0.12	70.00	196.00		34
H114	400	0.12	46.67	178.00		48
H115	200	0.16	32.00	155.33	116	104
H116						
H117	100	0.11	10.67	192.67		57
H118	200	0.23	46.67	163.00	51	251
H119	400	0.12	49.33	198.00		51
H120	400	0.12	46.67	209.00		26
H121	200	0.45	90.00	154.00	112	514
H122	200	0.12	24.00	171.00		63
H123	200	0.11	22.67	180.67		46
H124	200	0.10	20.00	212.33		53
H125	200	0.18	35.33	141.67	78	96
H126	200	0.15	30.67	138.00	63	108
H127	200	0.27	53.33	120.33	62	170
H128	200	0.16	32.00	136.00	109	87
H129	600	0.11	64.00	195.67		23
H130	400	0.18	70.67	235.33	130	103
H131	600	0.11	63.00	168.00		20
H132	600	0.11	68.00	160.00		32
H133	200	0.12	24.00	256.67		53
H134	200	0.12	24.67	139.67		63
H135	200	0.13	26.00	167.33		74
H136	400	0.13	50.67	161.33		42
H137	200	0.13	25.33	205.67	110	124
H138	200	0.21	41.33	140.67	62	152
H139	600	0.11	64.00	175.67		28
H140	400	0.15	58.67	112.00	161	70
H141	400	0.12	48.00	215.00		11
H142	400	0.11	44.00	164.33		33
H143	400	0.16	62.67	247.33	52	109
H144	200	0.23	46.67	127.67	61	161
H145	400	0.11	44.00	263.00		36
H146	200	0.24	48.67	147.00	78	213
H147	200	0.16	32.00	134.67	73	155
H148	200	0.13	26.67	170.67	44	80
H149	400	0.12	46.67	207.00		47
H150	200	0.16	31.33	155.67	131	160
H151	200	0.15	30.67	136.00	112	122
H152	400	0.12	46.67	161.67		43
H153	400	0.11	45.33	139.33		38
H154	400		48.00	244.00		33

Canine plasma						
S	Dil.	$n \times 10^9$/mL	corr $n \times 10^9$/mL	D_h(nm)	W(nm)	$N_{tracked}$
C1	50	1.21	60.50	138.67		
C2	50	0.89	44.33	179.33		
C3	1	1.83	1.83	293.00	179	601
C4	10	0.51	5.07	190.00	106	121
C5	50	2.47	123.67	135.33	53	842
C6	10	0.73	7.30	121.00	113	151
C7	50					
C8	50	0.82	40.83	130.00	53	238
C9	50	0.88	43.83	174.67	87	185
C10	50	0.43	21.67	122.67	76	123
C11	50	1.49	74.33	111.33	49	503
C12	50	1.16	58.00	123.33	85	381
C13	1	1.85	1.85	342.33	203	866
C14	50	0.58	29.00	133.33	128	157
C15	1				80	118
C16	50	1.33	66.50	206.33	104	553
C17	2	3.07	6.13	196.00	151	1065
C18	50	0.61	30.67	118.33	72	488
C19	50	0.63	31.50	123.67	89	353
C20	1	0.72	0.72	356.33	164	536
C21	50	1.76	87.83	128.33	71	1010
C22	50	0.28	14.00	162.67	138	137
C23	10	0.83	8.27	157.00	100	613
C24	50	1.26	63.00	127.33	82	891
C25	50	2.19	109.33	160.33	100	1053
C26	50	2.30	115.00	131.00	72	969

Canine blood						
S	Dil.	$n \times 10^9$/mL	corr $n \times 10^9$/mL	D_h(nm)	W(nm)	$N_{tracked}$
C1	200	0.18	36.00	177.67		
C2						
C3						
C4						
C5	500	0.30	150.00	147.33		54
C6	400	0.24	94.67	152.67		21
C7	400	0.94	377.33	164.00	118	354
C8	400	0.39	154.67	179.33	109	83
C9						
C10	600	0.21	128.00	148.00		40
C11	600	0.29	172.00	186.33		51
C12	600	0.16	98.00	158.33		35
C13	600					48
C14	600	0.78	470.00	191.67	161	234
C15						
C16	1000	0.11	110.00	157.67		25
C17	400	0.12	46.67	248.00		44
C18	1200	0.25	296.00	191.33	144	206
C19	400	0.21	84.00	171.00	135	120
C20	400	0.19	77.33	245.67	225	137
C21	400	0.16	62.67	120.33	153	82
C22	200	0.12	23.00	188.00		21
C23	400	0.10	41.33	210.67		23
C24	200	0.16	31.33	152.67	120	102
C25	200	0.42	83.33	159.33	108	528
C26	200	0.21	42.67	154.33	206	229

C27	50	2.09	104.33	123.00	64	947
C28	50	1.21	60.50	138.67	129	954
C29	50	0.62	31.00	126.33	109	424
C30	50	2.53	126.33	130.33	65	972
C31	50	1.89	94.33	130.67	62	980
C32	10	0.85	8.53	193.33	154	834
C33	50	1.00	49.83	142.33	77	737
C34	20	0.69	13.73	179.67	140	610
C35	20	0.47	9.47	124.00	93	415
C36	20	1.03	20.53	157.67	111	724
C37	20	1.28	25.67	126.33	76	776
C38	50	0.86	43.00	129.67	66	668
C39	50	1.32	66.00	137.67	91	949
C40	50	0.92	45.83	128.67	51	720
C41	50	1.16	58.00	138.33	66	869
C42	20	1.30	25.93	151.33	89	940
C43	20	1.55	31.07	140.33	89	974
C44	50	1.46	73.00	138.67	68	945
C45	200	1.41	281.33	132.67	66	944
C46	50	0.59	29.33	137.67	99	443
C47	50	2.76	137.83	129.33	48	1078
C48	50	0.56	28.00	158.00	76	577
C49	50	1.15	57.50	177.33	81	766
C50	20	0.64	12.73	166.67	125	652
C51	20	0.73	14.53	146.67	59	570
C52	20	0.62	12.47	169.67	93	415
C53	50	1.57	78.67	136.67	74	959
C54	20	1.39	27.80	185.67	144	965
C55	5	0.67	3.37	232.67	152	538
C56	20	0.65	13.00	207.67	179	446
C57	50	1.53	76.67	162.67	107	982
C58	20	0.97	19.47	196.00	130	812
C59	20	0.63	12.53	192.67	152	513
C60	20	0.68	13.53	186.67	172	477
C61	50	2.19	109.33	148.67	105	956
C62	20	0.85	17.07	177.67	136	654
C63	50	0.58	28.83	146.00	93	425
C64	50	0.60	29.83	127.00	108	475
C65	20	1.93	38.67	230.67	147	847
C66	50	1.17	58.50	143.00	66	254
C67	5	1.77	8.87	157.00	139	927
C68	20	1.39	27.87	141.33	93	881
C69	5	0.68	3.38	286.00	150	464
C70	2	0.85	1.71	321.33	177	661
C71	5	0.64	3.18	281.00	135	443
C72	50	0.45	22.33	237.67	146	301
C73	20	0.63	12.67	199.67	129	480
C74	20	0.91	18.27	183.00	88	650
C75	20	1.05	21.07	201.00	114	863
C76	3	3.77	11.31	231.00	99	1084
C77	20	2.00	39.93	162.67	208	943
C78	20	9.64	192.87	195.33	143	1127
C79	20	1.52	30.33	130.33	83	932
C80	20	1.68	33.60	132.67	208	946
C81	50	0.75	37.50	104.67	76	863
C82	50	1.21	60.33	113.33	78	923
C83	50	0.22	11.00	174.33	130	256
C84	50	0.53	26.50	135.33	109	598
C85	50	0.45	22.50	142.33	152	521
C86	20	0.45	8.93	179.00	177	436
C87	50	3.16	157.83	120.33	83	1073
C88	20	0.67	13.47	128.00	120	807
C89	10	0.67	6.67	207.00	172	824

C27	400	0.21	82.67	131.67	170	107
C28	200	0.18	36.00	177.67	155	131
C29	400	0.16	62.67	131.67	228	90
C30	200	0.28	55.33	137.00	92	277
C31	200	0.51	102.00	156.33		218
C32	200	0.14	27.33	187.33	79	79
C33	200	0.34	68.67	124.00	54	303
C34	200	0.18	35.33	167.33	59	77
C35	400	0.11	45.07	155.33		47
C36	600	0.13	80.00	191.33		41
C37	400	0.13	50.67	127.67		34
C38	600	0.13	76.00	132.33		49
C39	200	0.18	36.00	159.67	191	141
C40	400	0.13	52.00	152.33		54
C41	200	0.14	28.67	142.33	69	105
C42	800	0.12	96.00	234.33		47
C43						
C44	200	0.16	32.00	133.67		55
C45	200	0.46	92.67	156.67	75	490
C46	200	0.14	28.00	161.00	63	71
C47	200	0.27	54.67	135.67	99	275
C48	200	0.16	31.33	166.33		60
C49	400	0.18	70.67	189.33		73
C50	400	0.11	45.33	160.67		34
C51	600	0.10	62.00	178.33		32
C52	600	0.16	96.00	215.33		67
C53	200	0.24	48.67	141.67	56	183
C54	400	0.13	52.00	224.33		42
C55	400	0.16	62.67	219.33		79
C56	400	0.12	49.33	199.33		42
C57	200	0.19	38.67	161.33	91	164
C58	400	0.14	54.67	252.00		52
C59	400	0.13	53.33	238.67		51
C60	400	0.10	40.00	204.67		16
C61	200	0.34	68.67	157.67	109	335
C62	200	0.64	128.67	173.33	90	535
C63	400	0.12	48.00	148.33		40
C64	400	0.15	61.33	158.67	38	85
C65	200	0.13	26.67	266.00		60
C66	200	0.16	32.00	170.33	164	124
C67	400	0.14	57.33	179.67	40	92
C68	400	0.12	49.33	234.67		34
C69	400	0.12	48.00	238.67	114	64
C70	400	0.26	105.33	404.67	282	354
C71	600	0.10	58.40	214.00		8
C72	600	0.11	68.00	284.67		39
C73	1000	0.15	153.33	218.67	155	116
C74	800	0.11	88.00	317.33		38
C75						
C76						
C77						
C78						
C79						
C80						
C81	20	1.82	36.40	167.67	141	947
C82	20	0.46	9.13	123.67	111	678
C83	20	0.25	5.07	160.67	166	392
C84	20	0.24	4.80	138.67	111	313
C85	20	0.64	12.73	140.00	119	852
C86	20	0.10	2.00	163.33	164	131
C87	20	4.16	83.13	131.33	66	1069
C88	20	0.25	5.00	148.00	133	389
C89	20	0.20	3.93	198.00	151	260
